# Investigating GSK-3β as a Potential Prognostic Marker for Metastasis in Head and Neck Squamous Cell Carcinoma: A Preliminary Study on the Crossroads of GABAergic and Wnt Signaling

**DOI:** 10.1055/s-0045-1814075

**Published:** 2026-01-20

**Authors:** Omar Shebli Museedi, Bashar Hamid Abdullah, Natheer Hashim Al-Rawi

**Affiliations:** 1 Department of Oral Diagnostic Sciences, College of Dentistry, University of Baghdad, Baghdad, Iraq; 2 Department of Oral and Craniofacial Health Sciences, College of Dental Medicine, University of Sharjah, Sharjah, United Arab Emirates

**Keywords:** Carcinoma, squamous cell of head and neck, biomarker validation, gamma-aminobutyric acid, Wnt signaling pathway, beta-catenin, glycogen synthase kinase 3 beta, neoplasm metastasis, metastatic risk

## Abstract

**Objectives:**

The purpose of this study was to determine whether glycogen synthase kinase-3β (GSK-3β) mediates the link between γ-aminobutyric-acid (GABA) signaling and Wnt/β-catenin activation in the progression of head and neck squamous cell carcinoma (HNSCC) and to evaluate whether tumor GSK-3β expression can predict cervical nodal metastasis.

**Materials and Methods:**

Forty patients diagnosed with primary HNSCC supplied paired specimens of normal, dysplastic, and tumor tissues. Immunohistochemistry was performed for GABA-BR1/2, β-catenin, and GSK-3β. Nonparametric tests, Spearman's correlation, and multivariable logistic regression (adjusted for age, sex, T-stage, and site) were utilized to investigate clinicopathological associations. Receiver operating characteristic analysis was applied to evaluate the predictive performance for metastasis.

**Results:**

The expression of GABA-BR1/2 and nuclear β-catenin increased progressively from normal mucosa to dysplasia to carcinoma (all *p* < 0.001). A tumor GSK-3β score ≥ 6 independently predicted nodal metastasis after adjusting for standard clinicopathological variables (adjusted odds ratio = 8.8, 95% confidence interval: 1.9–39.6; *p* = 0.005), with an area under the curve (AUC) of 0.84. While promising, this AUC should be interpreted cautiously due to the small sample size. Multivariate analysis confirmed functional interactions between the GABAergic and Wnt pathways.

**Conclusion:**

GSK-3β appears to integrate GABAergic and Wnt/β-catenin signaling and may serve as a robust, independent biomarker of metastatic risk in HNSCC. Although preliminary, these findings support the potential clinical value of GSK-3β and warrant validation in larger, multicenter cohorts before considering its incorporation into risk stratification models or targeted therapeutic strategies.

## Introduction

Head and neck squamous cell carcinoma (HNSCC) remains a global health challenge due to its aggressive metastatic behavior, which often leads to poor patient outcomes. 1 2 Although surgery, radiotherapy, and chemotherapy provide partial therapeutic benefits, the overall survival rate for advanced HNSCC remains low. 1 A deeper molecular understanding of tumor progression and metastasis is crucial for identifying novel prognostic markers. 3 While various proteins have been explored in this journal to predict invasion, 4 a definitive molecular signature is still needed.

HNSCC represents a heterogeneous group of malignancies characterized by marked molecular complexity and therapeutic resistance. Despite advances in targeted therapies, such as epidermal growth factor receptor inhibitors and immune checkpoint blockade, the 5-year survival rate for patients with advanced disease remains below 50%, 5 underscoring the need for new molecular drivers and actionable pathways involved in HNSCC progression and metastasis. 6

Two key signaling axes—GABAergic (via GABA-B receptors [GABA-BR1/2]) and Wnt/β-catenin—have garnered attention for their roles in tumorigenesis. 7 Gamma-aminobutyric acid (GABA), primarily known as an inhibitory neurotransmitter in the central nervous system, 8 also modulates peripheral cancers, potentially affecting proliferation, migration, and apoptosis. 9 GABA signaling involves complex regulatory mechanisms through both ionotropic (GABA_A) and metabotropic (GABA_B) receptors, with widespread distribution beyond neural tissues. 10 In contrast, Wnt/β-catenin pathway dysregulation—evidenced by β-catenin accumulation in the cytoplasm and nucleus 11 12 —is a well-established contributor to many carcinomas, including HNSCC. 13 14 Aberrant activation of the Wnt/β-catenin signaling pathway is a hallmark of HNSCC. 13

While GABA signaling has traditionally been studied in neurological contexts, emerging evidence suggests that GABAergic pathways play crucial roles in nonneuronal tissues, including cancer. Recent studies have demonstrated that alterations in GABA-B receptor expression correlate with tumor aggressiveness in several malignancies, including breast, prostate, and colorectal cancers. 15 16 Some epithelial cancers can even acquire GABAergic properties resembling neural cells, particularly in metastatic settings, suggesting a potential role for GABA signaling in adaptive responses during cancer progression. 17 Notably, previous studies have identified GABA-B receptors as key components of cancer biology, further supporting investigation of this pathway in HNSCC. However, the role of GABAergic signaling in HNSCC pathogenesis remains largely unexplored.

Glycogen synthase kinase-3β (GSK-3β), traditionally considered a negative regulator of β-catenin, 12 has shown paradoxical prometastatic roles in certain cancers. 18 19 While GSK-3β displays complex, context-dependent functions in oncology, a comprehensive review has consolidated evidence of its protumorigenic activity in several malignancies, including HNSCC, providing a strong rationale for this investigation. 20 However, multiple studies have also reported tumor-suppressive effects, leading to its characterization as a “good cop, bad cop” molecule in cancer biology. 21 Addressing this conflicting evidence is critical to understanding its role in HNSCC.

The role of GSK-3β in HNSCC—especially regarding the interplay between GABAergic signaling and Wnt/β-catenin pathways—remains poorly defined. Although the individual roles of GABA signaling and Wnt dysregulation in cancer are established, their potential interaction through GSK-3β during HNSCC metastasis has not been fully elucidated. We therefore hypothesized that GSK-3β may act as a molecular “switchboard” integrating GABAergic and Wnt signals to enhance metastatic potential.

In light of the clinical need for actionable biomarkers in metastatic HNSCC, we investigated GSK-3β because of its known function as a central regulatory kinase influencing multiple substrates in key metastatic pathways, proposing it as a potential therapeutic target and prognostic marker. This study aimed to characterize the expression patterns and subcellular localization of GABA-B receptors (GABA-BR1/2), β-catenin, and GSK-3β across the progression of HNSCC (normal mucosa, dysplasia, and invasive carcinoma) using immunohistochemistry (IHC), and to evaluate their intercorrelations and relationships with clinicopathological features, particularly metastatic status, to identify potential biomarkers and therapeutic targets within this novel signaling network.

## Materials and Methods

A cohort of 40 patients with primary HNSCC treated at a university teaching hospital between January 2021 and December 2022 was analyzed. The research complied with the Declaration of Helsinki and received Institutional Review Board approval (protocol no. 742, approved on December 1, 2022). All procedures adhered to institutional guidelines, and written informed consent was obtained from all participants. Out of 45 screened patients, five were excluded—two due to incomplete records and three due to inadequate tissue. Clinicopathological data, including the 12-month metastatic status verified through radiological (computed tomography/magnetic resonance imaging) or histopathological methods, were documented ( Table 1 ). This study on tumor marker prognosis was conducted and reported in accordance with the REMARK (Reporting Recommendations for Tumor Marker Prognostic Studies) guidelines.

**Table 1:** Demographic and clinicopathological characteristics of the study cohort

Characteristic	*N* (%)
**Gender**	
Male	28 (70)
Female	12 (30)
**Age**	
Mean ± SD	63.0 ± 10.4
Range	42–78
**Anatomical** s **ite**	
Larynx	13 (32.5)
Tongue	10 (25.0)
Floor of mouth	6 (15.0)
Buccal mucosa	5 (12.5)
Other	6 (15.0)
**Tumor** s **tage**	
Stage I	5 (12.5)
Stage II	8 (20.0)
Stage III	12 (30.0)
Stage IV	15 (37.5)
**Metastasis** s **tatus**	
Metastatic	18 (45.0)
Non-metastatic	22 (55.0)
**Perineural** i **nvasion**	
Present	14 (35.0)
Absent	26 (65.0)

Abbreviation: SD, standard deviation.

IHC was performed on 4-μm paraffin-embedded tissue sections. Following deparaffinization, rehydration, and antigen retrieval (citrate buffer pH 9.0, water bath, 20 minutes), sections were blocked for endogenous peroxidase (3% H _2_ O _2_ ) and incubated overnight at 4°C with primary antibodies against GABA-BR1 (1:500, Abcam, Cat# ab238130), GABA-BR2 (1:100, Abcam, Cat# ab75838), β-catenin (Ready-to-Use, Dako, Cat# GA702), and GSK-3β (1:4000, Abcam, Cat# ab93926). Antibody specificity was confirmed according to manufacturer datasheets using recommended positive controls (cerebral cortex for GABA-BR1/2, colon for β-catenin, and placenta for GSK-3β), which showed expected staining patterns, while negative controls (primary antibody omission) showed no staining. Optimized dilutions, positive controls, and negative controls ensured assay validity. Detection utilized a biotinylated secondary antibody, streptavidin-horseradish peroxidase, and 3,3'-diaminobenzidine (DAB) chromogen, with hematoxylin counterstaining.

Following IHC, slides were coded to blind the investigators to clinicopathological data. A hybrid scoring approach was employed to enhance objectivity. For the quantitative assessment of staining intensity, five representative, nonoverlapping high-power fields (400× magnification) were captured as digital images. Using ImageJ software (National Institutes of Health, United States) with the color deconvolution plugin, the mean optical density of the isolated DAB (brown) stain was calculated and assigned an intensity score (0 = negative, 1 = weak, 2 = moderate, 3 = strong). Concurrently, the percentage of positively stained tumor cells within the same fields was semiquantitatively evaluated by two independent, blinded pathologists and assigned a score (e.g., 0 = 0%, 1 = 1–25%, etc.). The final H-score for GABA-BR1/2 was calculated by multiplying the quantitative intensity score by the pathologist-derived percentage score. 22 For β-catenin and GSK-3β, the final additive score was the sum of the intensity and percentage scores. 23 High interobserver agreement for the final calculated scores was confirmed (Cohen's kappa = 0.85), validating the reliability of this hybrid method.

Statistical analysis was conducted using IBM SPSS v26 and R v4.1.2, with a significance threshold of *p* < 0.05 (two-tailed tests).

An *a priori* power analysis demonstrated 80% power to detect an effect size of 0.45 ( *α* = 0.05) for comparing GSK-3β expression between metastatic and nonmetastatic groups with *n* = 40.

Because data were nonnormally distributed (Shapiro–Wilk test), the Mann–Whitney *U* and Kruskal–Wallis tests with Dunn's post hoc analysis were applied for group comparisons.

Spearman's correlation evaluated associations between markers.

Univariate and multivariate logistic regression examined relationships between marker expression (using the optimal GSK-3β cutoff determined by receiver operating characteristic [ROC] analysis) and metastasis, while controlling for age, sex, stage, and site. Odds ratios (ORs) and 95% confidence intervals (CIs) were reported.

Pathway interactions were analyzed using principal component analysis (PCA) and hierarchical clustering based on Euclidean distance and Ward's D ^2^ linkage.

Bonferroni correction was applied for multiple comparisons.

A complete case approach was used, as missing data were < 5%.

Model robustness was validated through 1,000 replicate bootstrap resampling.

## Results

### Marker Expression Profiles

**Fig. 1A:**
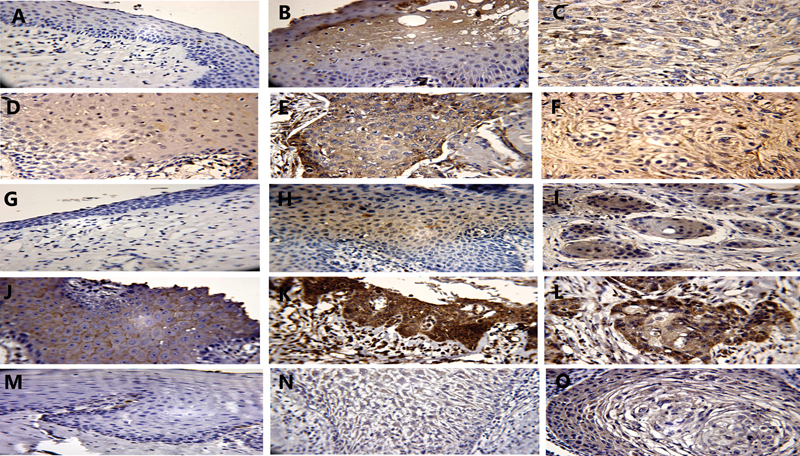
Immunohistochemical Staining of GABAergic Markers, β-Catenin, and GSK-3β in HNSCC. Representative micrographs illustrating protein expression in normal mucosa (N), dysplastic epithelium (D), and tumor tissue (T). Magnification: 400×; Scale bar: 50 μm. Brown DAB staining indicates positive immunoreactivity. ( – **C** ) GABA-B Receptor (GABA-B): Predominantly cytoplasmic staining with increased expression in dysplastic tissue. ( **D** – **F** ) GABA-B Receptor 1 (GABA-BR1): Membranous and cytoplasmic immunoreactivity, with markedly higher intensity in dysplasia and tumor than in normal mucosa. ( **G** – **I** ) GABA-B Receptor 2 (GABA-BR2): Progressive cytoplasmic and nuclear staining from normal to dysplasia to carcinoma. ( **J** – **L** ) β-Catenin (β-Cat): Transition from membranous localization in normal tissue to cytoplasmic and nuclear accumulation in tumor tissue, consistent with Wnt pathway activation. ( **M** – **O** ) Glycogen Synthase Kinase-3β (GSK-3β): Variable cytoplasmic expression across tissue types, with enhanced staining intensity in dysplastic and tumor specimens.

Immunohistochemical evaluation demonstrated distinct expression patterns among tissue types, as illustrated in representative micrographs ( Fig. 1 ). Significant disparities were noted in the subcellular localization of β-catenin and the intensity of GSK-3β staining when contrasting tumor tissues with normal mucosa.

GABA-BR1/2: Both subunits exhibited significant overexpression in dysplastic and malignant tissues relative to normal tissues, with peak GABA-B expression observed in dysplasia.

β-catenin: A distinct shift from membranous localization in normal tissues to cytoplasmic and nuclear localization in dysplastic and malignant tissues indicated activation of Wnt signaling. 14

GSK-3β: Tumor tissues demonstrated a marked increase in GSK-3β expression (median additive score = 8) compared to both normal (median = 2) and dysplastic tissues (median = 6) ( *p* < 0.001).

The expression of GABA-BR1 exhibited a substantial quantitative rise from normal tissues (median H-score = 2.0) to dysplastic tissues (median = 7.5, *p* < 0.001) and tumor tissues (median = 6.0, *p* < 0.001), as seen in Table 1 .

**Fig. 2:**
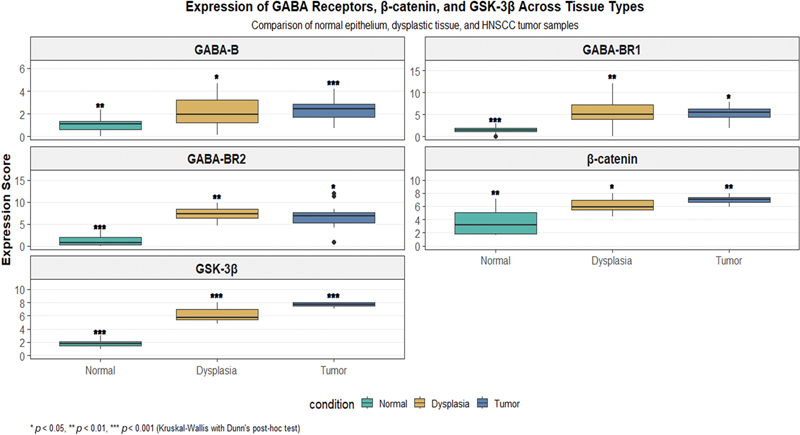
Box plots illustrate marker expression in normal, dysplastic, and tumor tissue. The expression levels of GABA-BR1, GABA-BR2, β-catenin, and GSK-3β show a progressive increase from normal to dysplastic to tumor tissues. Compared with normal tissues, dysplasia and tumor samples exhibit significant overexpression of GABA-BR1, GABA-BR2, and GSK-3β, with peak GABA-B receptor expression observed in dysplasia. β-catenin expression increases from normal to tumor, indicating cytoplasmic/nuclear accumulation during malignant progression. GSK-3β, glycogen synthase kinase-3β; GABA-BR1/2, GABA-B receptor subunits 1 and 2. * *p* < 0.05, ** *p* < 0.01, *** *p* < 0.001 using Kruskal–Wallis and Dunn's post hoc tests.

Overall, the expression of GABA-BR1, GABA-BR2, and cytoplasmic/nuclear β-catenin displayed a statistically significant progressive increase from normal to dysplastic and tumor tissues (all *p* < 0.001). Box plots ( Fig. 2 ) confirmed these trends; the presence of outliers suggests underlying molecular heterogeneity within the patient cohort, potentially reflecting biologically distinct subtypes warranting further study. Fig. 2 provides a visual summary of these differences in marker expression.

**Fig. 3:**
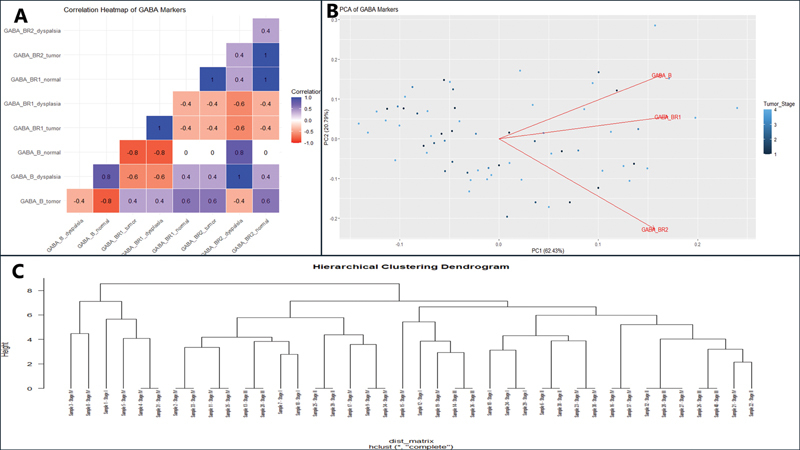
Multivariate marker expression analyses. ( **A** ) Relation heatmap shows considerable positive connections across tumor GABAergic markers and context-dependent expression changes from normal to malignant tissues. ( **B** ) Principal component analysis (PCA) plot (PC1 vs. PC2) reveals ∼83% variance, distinguishing normal from malignant tissues. ( **C** ) Hierarchical clustering dendrogram links GABAergic/Wnt markers to head and neck squamous cell carcinoma (HNSCC) development in higher stage tumors. PC1/2, principle components 1 and 2; HNSCC, head and neck squamous cell cancer.

The Spearman's correlation analysis, shown in the heatmap ( Fig. 3A ), revealed strong positive associations among GABAergic markers, particularly within tumor tissues (e.g., GABA-B_tumor vs. GABA-BR1_tumor: *ρ* ≈ 0.8). In contrast, negative correlation was observed between certain dysplastic/tumor GABA-B markers and their normal counterparts, underscoring a context-dependent shift during neoplastic transformation. GABA-BR1 expression exhibited a strong correlation with GABA-BR2 in tumor tissues ( *ρ* = 0.81, *p* < 0.001) and dysplastic tissues ( *ρ* = 0.74, *p* < 0.001), suggesting a coordinated regulatory mechanism. The expression of GSK-3β in tumor tissues demonstrated a moderate positive correlation with nuclear β-catenin ( *ρ* = 0.59, *p* < 0.001) and GABA-BR1 ( *ρ* = 0.47, *p* = 0.002), suggesting a potential pathway crosstalk. Together, these findings indicate coordinated regulation of GABAergic signaling components during the progression of HNSCC.

### Association of Studied Markers with Clinicopathological Features

GSK-3β expression exhibited a significant correlation with metastatic disease. Univariate logistic regression analysis indicated that GSK-3β overexpression (utilizing a suitable cutoff) serves as a significant predictor of metastasis (OR = 9.52, 95% CI: 2.15–42.17, *p* = 0.003). This notable association remained significant after controlling for potential confounders such as age, gender, tumor stage, and anatomical site in a multivariate logistic regression model (adjusted OR = 8.76, 95% CI: 1.94–39.58, *p* = 0.005), supporting the role of GSK-3β overexpression as an independent predictor of metastasis in this patient cohort. ROC analysis indicated strong discriminatory capability (area under the curve [AUC] = 0.84, 95% CI: 0.72–0.96), with a sensitivity of 83% and specificity of 77% at a score threshold of ≥ 6 ( Fig. 3B ). The data suggest a substantial predictive capability of increased GSK-3β expression for HNSCC metastasis. In contrast to the strong findings for GSK-3β, no significant associations were found between either GABA receptor or β-catenin expression and lymph node metastasis or tumor T stage after Bonferroni correction.

**Fig. 4:**
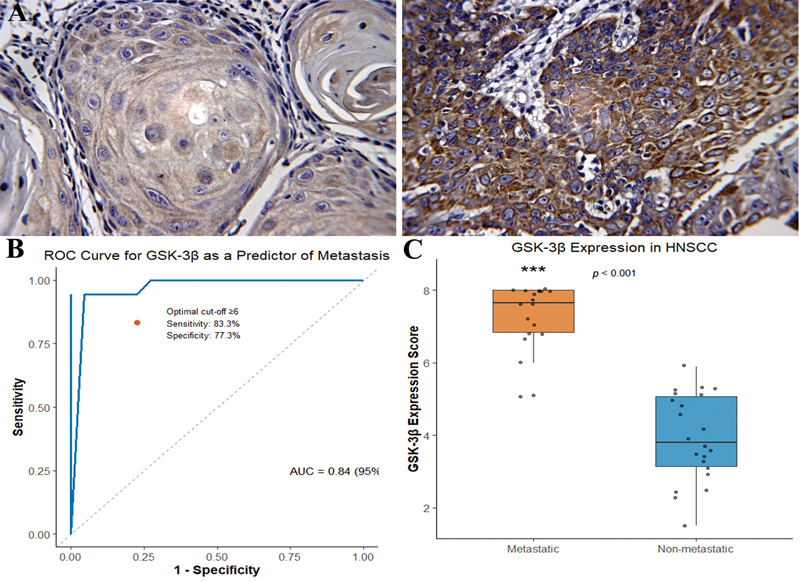
GSK-3β Expression and Metastatic Status in HNSCC. ( **A** ) Immunohistochemical images showing higher cytoplasmic GSK-3β staining in metastatic vs. non-metastatic tumors (400×, scale bar: 50 μm). ( **B** ) Box plot demonstrating significantly elevated GSK-3β scores in metastatic tumors (median 7.5 vs. 4.0; *p* < 0.001). ( **C** ) ROC curve showing good predictive performance of GSK-3β for metastasis (AUC 0.84; optimal cutoff ≥6).

A comparative analysis of metastatic and nonmetastatic tumors ( Fig. 4 ) revealed significantly elevated GSK-3β expression in metastatic samples (median = 7.5 vs. 4.0, *p* < 0.001), thereby reinforcing its potential as a biomarker for HNSCC aggressiveness.

## Multivariate Analyses (PCA and Clustering)

As an exploratory analysis to visualize patterns in the data, PCA of marker expression data, as visualized in Fig. 3B , revealed two principal components (PC1 and PC2) that together explained 83.2% of the total variance (PC1: 61.5%, PC2: 21.7%). PC1 strongly separated normal (left cluster) from dysplastic and tumor tissues (right cluster). High loadings for GABA-B, GABA-BR1, and GABA-BR2 on PC1 underscored the importance of GABAergic components in distinguishing normal from neoplastic tissues

Hierarchical clustering analysis identified three major clusters ( Fig. 3C ): cluster 1 consisted predominantly of normal tissues (86.7%), cluster 2 comprised a mix of dysplastic tissues (75.0%) and early-stage tumors (25.0%), while cluster 3 contained primarily advanced stage tumors (93.3%, stages III–IV). This clustering pattern suggests that the combined GABAergic/Wnt expression profile correlates strongly with disease progression and may hold promise as a molecular staging tool.

## Discussion

The findings suggest a potential molecular interaction between GABAergic signaling and the Wnt/β-catenin pathway in the progression of HNSCC, identifying GSK-3β as a crucial nexus and an independent predictor of metastasis. These results enhance our understanding of HNSCC biology and highlight GSK-3β as a potential therapeutic target and biomarker for aggressive disease.

These findings support a model in which GABAergic signaling and Wnt/β-catenin pathways converge to promote HNSCC progression. GABA-B receptor upregulation in dysplasia may prime tissues for malignant transformation, while sustained GABA-BR1/2 expression in later stages aligns with tumor growth and invasion. The shift in β-catenin from membrane bound to cytoplasmic/nuclear compartments further emphasizes canonical Wnt pathway activation. 14 Collectively, these expression patterns suggest a coordinated dysregulation of GABAergic and Wnt/β-catenin signaling during HNSCC tumorigenesis.

The peak of GABA-B receptor expression in dysplastic tissues suggests that GABAergic signaling alterations may represent an early event in HNSCC pathogenesis, potentially contributing to initial neoplastic transformation. This finding aligns with recent studies in other malignancies where GABAergic pathway dysregulation has been implicated in early carcinogenesis. 21 24 The subsequent moderate decrease in GABA receptor expression in established tumors compared to dysplastic tissues suggests dynamic regulation during disease progression, possibly reflecting adaptation to changing microenvironmental conditions or selective pressures during malignant evolution.

GSK-3β overexpression strongly correlated with metastasis, suggesting a prometastatic role in HNSCC. This finding aligns with the context-dependent oncogenic functions of GSK-3β summarized by Domoto et al, 20 and our results extend this understanding by demonstrating a strong, independent association with metastatic potential in HNSCC. This duality of function has been termed the “good cop, bad cop” nature of GSK-3β in cancer biology, with context-dependent tumor-suppressive or oncogenic roles. 25 While canonically a tumor suppressor via β-catenin degradation, its prometastatic function in our cohort may depend on specific posttranslational modifications or interactions with noncanonical pathways that override its tumor-suppressive effects. These data highlight GSK-3β as a promising biomarker for HNSCC aggressiveness. Although canonically a β-catenin antagonist, its multifaceted functions vary by tumor context, warranting further mechanistic investigation.

The identification of GSK-3β as an independent predictor of metastasis (adjusted OR = 8.76) is significant, given the challenges of identifying reliable biomarkers for HNSCC progression. Our findings align with other biomarker studies reporting progressive expression changes of proteins, such as METTL3 and E-cadherin, from normal to malignant oral tissues. 26 27 In this context, GSK-3β represents a novel marker that not only fits this progressive pattern but also integrates key oncogenic signaling pathways. Its association remained significant after adjusting for standard clinicopathological factors, suggesting that GSK-3β overexpression may represent a fundamental biological process in metastatic transformation rather than a secondary consequence. The AUC value of 0.84 supports its potential clinical utility as a predictive biomarker.

**Fig. 5:**
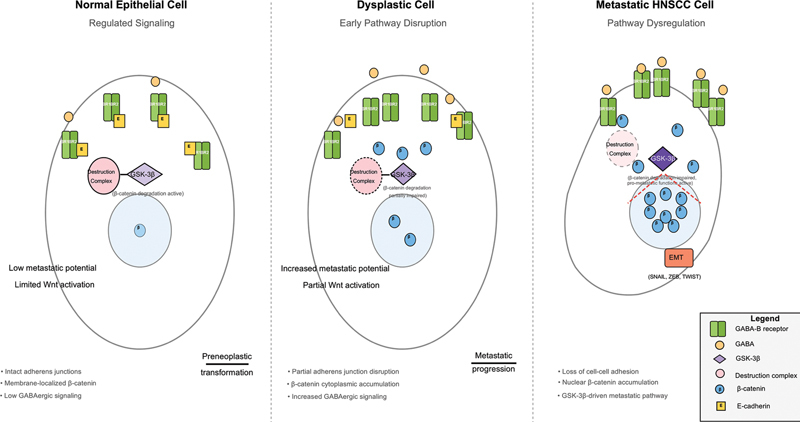
Proposed hypothetical model of GABAergic-Wnt pathway crosstalk via GSK-3β in head and neck squamous cell carcinoma (HNSCC) progression. This schematic illustrates the proposed molecular interplay between GABAergic and Wnt signaling. In normal epithelium, GSK-3β actively promotes β-catenin degradation. In dysplastic tissue, we hypothesize that increased GABA-B receptor signaling leads to the activation of second messenger pathways (e.g., PI3K/AKT), resulting in the inhibitory phosphorylation of GSK-3β. This modulation causes partial β-catenin stabilization, an early event in tumorigenesis. In metastatic HNSCC, we propose that sustained GABAergic signaling further impairs β-catenin degradation and allows GSK-3β to act on other prometastatic substrates, promoting pathways like epithelial-mesenchymal transition (EMT). This proposed dysregulation drives metastatic potential. This model is hypothetical, based on the correlational findings of this study, and requires further mechanistic and functional validation.

Several mechanisms may explain the paradoxical prometastatic role of GSK-3β in HNSCC. It may selectively interact with alternative substrates within the tumor microenvironment, such as transcription factors driving epithelial-mesenchymal transition (EMT). 18 GSK-3β may also engage in noncanonical pathways, such as nuclear factor kappa B signaling, thereby enhancing cell survival and invasion. 28 Moreover, posttranslational modifications of GSK-3β could alter substrate specificity, redirecting GSK-3β from β-catenin degradation to prometastatic pathways. 29 The proposed model ( Fig. 5 ) illustrates this potential interplay, hypothesizing that GABA-B receptor activation could lead to second messenger signaling (e.g., via PI3K/AKT pathways) which in turn modulates GSK-3β phosphorylation at key inhibitory sites. This proposed mechanism remains speculative and requires direct functional validation.

The significant association of GSK-3β with metastatic risk underscores its potential as a therapeutic target. Future clinical trials exploring GSK-3β inhibitors—alone or combined with current therapies—are warranted. For instance, 9-ING-41 has shown antitumor activity in refractory tumors. 30 Similarly, tideglusib has shown efficacy in preclinical glioblastoma models and is currently under investigation for various cancers. 31 32 However, systemic inhibition of this ubiquitous kinase may lead to on-target toxicities, such as liver toxicity observed in prior trials. This underscores the need for selective inhibitors or targeted delivery systems to improve safety and efficacy. Our data provide a strong rationale for exploring these agents in HNSCC, particularly in advanced or metastatic disease.

While this study provides novel insights and robust clinical associations, several limitations must be acknowledged. First, although our power analysis demonstrated statistical adequacy for the primary endpoint, the cohort size ( *n* = 40) remains modest for biomarker validation. Thus, larger, independent, multicenter prospective studies with extended follow-up periods are essential to confirm GSK-3β's predictive robustness, generalizability, and prognostic significance in relation to disease-free and overall survival.

Second, the correlative nature inherent to immunohistochemical analyses in human tissues underscores the need for mechanistic validation. Future research should conduct functional studies using HNSCC cell lines, patient-derived organoids, and *in vivo* models to manipulate GABA-B and GSK-3β expression or activity directly, assessing consequent impacts on metastatic processes such as migration, invasion, EMT, and Wnt signaling dynamics.

Third, intratumoral heterogeneity presents another critical consideration. Although careful scoring was performed on representative areas, advanced techniques like single-cell ribonucleic acid sequencing and spatial transcriptomics could further delineate cell type-specific interactions, enhancing our understanding of tumor complexity and pinpointing subpopulations primarily driving metastasis.

## Limitations of the Study

While this study provides novel insights and robust clinical associations, several limitations must be acknowledged. First, the modest cohort size ( *n* = 40) renders these findings preliminary and hypothesis-generating rather than confirmatory. Second, the correlative nature of immunohistochemical data necessitates mechanistic validation through *in vitro* and *in vivo* models assessing GABA-B/GSK-3β manipulation and its impact on migration, invasion, EMT, and Wnt signaling. Third, potential confounders such as smoking history and human papillomavirus status were not available and should be included in future analyses. Finally, although interobserver agreement was high, the semiquantitative nature of IHC scoring introduces inherent subjectivity.

## Conclusion

This research provides preliminary evidence for a novel interaction between GABAergic signaling and Wnt pathways mediated by GSK-3β, suggesting its role as a significant, independent biomarker for metastasis in HNSCC. This integrative approach underscores the potential clinical relevance of GSK-3β for risk stratification and targeted therapy.

These findings provide a strong rationale for future translational and mechanistic studies aimed at improving therapeutic outcomes for patients with metastatic HNSCC.
